# The effect of dual-task conditions on postural control in adults with low back pain: a systematic review and meta-analysis

**DOI:** 10.1186/s13018-023-04035-6

**Published:** 2023-08-01

**Authors:** Mohammadreza Pourahmadi, Hossein Negahban, Bart Willem Koes, César Fernández‐de‐Las‐Peñas, Ismail Ebrahimi Takamjani, Mehrdad Bahramian

**Affiliations:** 1 Department of Physical Therapy, School of Paramedical and Rehabilitation Sciences, Mashhad University of Medical Sciences, Mashhad, Iran; 2 Rehabilitation Research Center, Department of Physiotherapy, School of Rehabilitation Sciences, Iran University of Medical Sciences, Tehran, Iran; 3 Orthopedic Research Center, Mashhad University of Medical Sciences, Mashhad, Iran; 4 Department of General Practice, Erasmus MC, University Medical Center, Rotterdam, The Netherlands; 5 Research Unit of General Practice, Department of Public Health and the Center for Muscle and Joint Health, University of Southern Denmark, Odense, Denmark; 6 Department of Physical Therapy, Occupational Therapy, Rehabilitation and Physical Medicine, Universidad Rey Juan Carlos (URJC), Alcorcón, Madrid, Spain; 7 Department of Physical Therapy, College of Health Science & Professions, University of North Georgia, Dahlonega, USA

**Keywords:** Dual task, Postural balance, Center of pressure, Low back pain, Systematic review

## Abstract

**Background:**

Dual-task conditions, which involve performing two tasks simultaneously, may exacerbate pain and further impair daily functioning in individuals with low back pain (LBP). Understanding the effects of dual-task conditions on postural control in patients with LBP is crucial for the development of effective rehabilitation programs. Our objective was to investigate the impact of dual-task conditions on postural control in individuals with LBP compared to those without LBP.

**Methods:**

We conducted a comprehensive search of Medline via PubMed, Scopus, the Cochrane Central Register of Controlled Trials, Web of Science, and EMBASE databases, with no language restrictions, from inception to January 1, 2023. The primary outcome measures of the study were velocity, area, amplitude, phase plane portrait, and path/sway length of the center of pressure (CoP). Standardized mean difference (SMD) effect sizes were calculated, and the quality of the studies was assessed using the Newcastle–Ottawa Scale (NOS).

**Results:**

From 196 studies, five involving 242 adults (≥ 18 years) met the inclusion criteria. Three studies were rated as high quality, while two were deemed moderate. In the included studies, 140 participants had non-specific LBP, while 102 participants did not report any symptoms, with mean ages of 36.68 (± 14.21) and 36.35 (± 15.39) years, respectively. Three studies had both genders, one exclusively included females, and one did not specify gender. Meta-analyses of primary outcomes revealed no significant differences in postural control between patients with LBP and pain-free controls during both easy and difficult postural tasks and cognitive load for velocity (easy: SMD − 0.09, 95% CI − 0.91 to 0.74; difficult: SMD 0.12, 95% CI − 0.67 to 0.91), area (easy: SMD 0.82, 95% CI − 2.99 to 4.62; difficult: SMD 0.14, 95% CI − 2.62 to 2.89), phase plane (easy: SMD − 0.59, 95% CI − 1.19 to 0.02; difficult: SMD − 0.18, 95% CI − 0.77 to 0.42), path/sway length (easy: SMD − 0.18, 95% CI − 0.77 to 0.42; difficult: SMD − 0.14, 95% CI − 0.84 to 0.55), and amplitude (easy: SMD 0.89, 95% CI − 1.62 to 3.39; difficult: SMD 1.31, 95% CI − 1.48 to 4.10).

**Conclusions:**

The current evidence suggests that there are no significant differences in postural control parameters during dual-task conditions between individuals with non-specific LBP and pain-free subjects. However, due to the limited number of available studies, significant publication bias, and considerable statistical heterogeneity, definitive conclusions cannot be drawn. Therefore, further research comprising high-quality studies with larger sample sizes is necessary to obtain conclusive results.

*Trial registration* PROSPERO CRD42022359263.

**Supplementary Information:**

The online version contains supplementary material available at 10.1186/s13018-023-04035-6.

## Introduction

Low back pain (LBP) is a global health issue and a significant cause of years lived with a disability [1]. This complex condition can lead to balance impairments and altered postural control [2–5]. Previous research has shown that young individuals with recurrent LBP tend to adopt a postural control strategy that involves stiffening their trunk and relying heavily on ankle proprioception to maintain their posture during quiet upright standing [4, 6]. These results indicate that trunk stiffening is primarily achieved through the co-contraction of trunk musculature, aided by augmented distal responses, in order to respond to unexpected perturbations in the support surface [7].

Postural control can be defined as the ability to control the body in space for two main purposes: balance and orientation [8]. Postural control is influenced by the interaction between the components including somatosensory, visual, and vestibular inputs [3]. As per the available literature, it has been reported that patients with LBP exhibit impaired somatosensory processing, including reduced tactile acuity [9], faulty muscle responses [3, 10], and trunk repositioning error [3, 10]. Consequently, this may lead to deficits in postural control and balance [2, 3], ultimately increasing the risk of falling and musculoskeletal injuries [9]. Additionally, Mazaheri et al. have highlighted that postural control impairments may also persist during the asymptomatic periods in which patients with LBP experience no pain [11]. The presence of these changes in the absence of a pain episode may be attributed to the fear of pain [11].

In postural control assessment, the dual-task concept is usually used to generate a more challenging situation [12]. A dual-task condition pertains to concurrent performance of two tasks that can be executed independently and have distinct and separate purposes [13]. In experimental setups involving dual-task conditions, the primary task is typically a static (*e.g.,* holding a yoga pose) or dynamic motor task (*e.g.*, running), while the secondary task is typically a cognitive task [12]. The interaction between postural and cognitive tasks depends on various factors such as the difficulty of the cognitive task, the difficulty of the postural task, and the integrity of sensorimotor/cognitive processes [14]. It has been observed that individuals with LBP exhibit a diminished ability to perform dual tasks compared to healthy individuals [15]. This can be attributed to the diversion of attentional and cognitive resources toward coping with pain, thereby leaving limited capacity for performing the other task. Such a reduction in performance can further affect daily activities [16], including driving while experiencing LBP. Furthermore, some studies have indicated that dual-task conditions requiring cognitive or motor processing can lead to decreased postural control and stability in individuals with LBP [14, 15]. However, the findings in the available literature are not entirely consistent [17]. For instance, a study suggested that when performing a dual task, the introduction of low attention loads impeded the conscious processing of postural memory associated with pain, resulting in improved trunk coupling compared to a single-task condition [5].

Given the conflicting findings and conclusions in previous studies, a well-designed systematic review and meta-analysis is warranted to identify the effect of dual-task conditions on postural control in individuals with LBP. By understanding the impact of dual-task conditions on postural control in LBP, healthcare professionals can develop more targeted rehabilitation plans for these individuals. To the best of our knowledge, this is the first systematic review and meta-analysis to specifically investigate the effects of dual-task conditions on postural control in individuals with LBP compared to those without LBP. The objective of this study was to assess the effect of dual-task conditions on postural control in individuals with LBP in comparison to individuals without LBP.

## Method

The methodology employed in this study adhered to the systematic review methods outlined in the Cochrane Handbook [18], and the findings were reported in accordance with the Preferred Reporting Items for Systematic Reviews and Meta-Analyses (PRISMA) 2020 statement [19]. The protocol for this systematic review has been registered in the international prospective register of systematic reviews (PROSPERO; registration number: #CRD42022359263).

### Eligibility criteria

The PECO (*i.e.,* Population; Exposure; Comparison; Outcome) framework [20] was used to develop the research question and to define the search terms and eligibility criteria for the systematic review.

Population: The included population comprised adults of both genders (≥ 18 years) with LBP. LBP was defined as pain occurring between the 12th ribs and the gluteal folds, with or without accompanying leg pain, lasting for a duration of at least one day [21]. No specific restrictions were placed on the type of LBP in our study. Exclusion criteria were age less than 18 years, psychological, mental, motor, or other physical disorders that could potentially influence postural stability [12].

Exposure: We included studies that met the following criteria: (1) the study protocol involved the evaluation of postural stability while participants were simultaneously performing a secondary task condition, and (2) participants performed the postural stability task in a standing posture [12]. No restrictions were placed on the assessment instruments utilized to collect data on postural control.

Comparison: The comparison should have been conducted with a control group, which was defined as participants without LBP.

Outcome: The prespecified primary outcomes were center of pressure (CoP) parameters, including mean velocity (cm/s), area (cm^2^), amplitude (cm), phase plane portrait, and path/sway length (cm^2^). A phase plane portrait in postural control refers to a graphical representation of the movement pattern of CoP over time during quiet standing. It is a two-dimensional plot that shows the CoP movement in the anterior–posterior direction on the x-axis and in the medial–lateral direction on the y-axis [14]. The phase plane portrait is used to analyze the stability and control of the CoP movements. The shape and orientation of the trajectory of CoP movement in the phase plane portrait can reveal important information about the coordination of postural control. Sway length of CoP is a measure of the overall distance covered by the CoP over a period of time [22]. We have chosen these specific outcomes as our primary outcomes because they are commonly utilized as parameters for assessing postural control. For studies to be included, they had to have at least one of the prespecified primary outcomes of this review. The secondary measure assessed was the reaction time, which refers to the duration from the moment the perturbation began to the initial response of the CoP as it lifted off from the baseline [15].

For the study design, all comparative observational, experimental, and quasi-experimental study designs were considered eligible for inclusion in this study. However, reviews, meta-analyses, conference proceedings, abstracts, editorials, opinions, books, letters, commentaries, and non-peer-reviewed journal articles were excluded from the analysis.

### Information sources

We searched the following electronic databases from their inception until 1 January 2023: Medline via PubMed, Scopus, the Cochrane Central Register of Controlled Trials, Web of Science, and EMBASE using the appropriate medical subject headings (MeSH), Thesaurus, and free-text terms. To develop the search strategy, we combined the P, E, and O components of the PECO framework by utilizing the AND operator. We deliberately chose not to include the C component in order to achieve a comprehensive search approach. For the P component, we combined terms such as "low back pain", "low back ache", "lumbago", "lumbalgia", and "back disorder", along with their relevant synonyms. Regarding the E component, we included terms like "dual task", "double task", "second tasks", "cognitive task", "double assignment", "cognitive load", “reaction time”, and "cognitive performance", along with their respective synonyms. For the O component, we incorporated terms such as "postural stability", "postural control", "postural equilibrium", "postural sway", "postural interference", and "center of pressure", alongside their corresponding synonyms. Synonym terms within each component were combined using the OR operator (see Additional file 1). The final search strategy was subjected to review by a library scientist following the PRESS (Peer Review of Electronic Search Strategies) guidelines [23]. Additional studies were identified by manual searching [17] and cross-references and contacting expert authors from the field.

We did not review content from file sources that originated from mainstream publishers, including ScienceDirect, Wiley, SAGE, Wolters Kluwer, and Taylor & Francis. Finally, there were no restrictions applied in terms of language, publication date, or publication status. The reporting of the literature search in this study was done using an extension of the PRISMA statement (PRISMA-S) [24].

### Study selection

The retrieved studies were imported into Endnote 20 software (Clarivate Inc., Philadelphia, PA, USA) and duplicates were removed using the EndNote deduplication feature. Moreover, some duplicates were checked manually due to variations in reference styles across electronic sources. Two authors (M.P. and M.B.) first independently screened the titles of the retrieved studies. Then, the same authors reviewed the abstracts of all potentially eligible studies. Finally, full-text studies meeting the criteria were selected and reviewed by both authors and evaluated for inclusion in the present review. Disagreements were settled by discussion or adjudication (H.N.).

### Data extraction

Two authors (M.P. and M.B.) independently extracted data and filled out predesigned forms. We used Microsoft Excel 365 spreadsheet software (Microsoft, Redmond, Washington, USA). Information including name of first author, year of publication, country, methodological quality, participant characteristics, setting, dual-task conditions, and outcomes. The extracted data were thoroughly reviewed for errors by a third author (H.N.).

### Quality assessment within individual studies

Pourahmadi et al. [20] noted that there is currently no established and validated tool for evaluating the risk of bias in comparative observational studies. Therefore, in our study, we utilized the Newcastle–Ottawa Scale (NOS) to appraise the quality of the studies that we included. The NOS is recommended by the Cochrane Non-Randomized Studies Methods Working Group to assess the quality of observational studies. The original scale, which is very comprehensive, is based on the following three subscales: Selection (4 items), Comparability (1 item), and Outcome or Exposure (3 items; [25]). Considering the objectives of this systematic review, the modified version of the NOS [25] was utilized for the reliability and validity assessment of the instruments used to evaluate postural control. Furthermore, aspects of the statistical analysis in the selected studies were also appraised, including sample size justifications and appropriateness and clarity of the statistical analysis method presentations [26]. Differences in age, gender, and physical activity were considered to explore the comparability subscale of the NOS [25, 26]. A total score of 3 or lower was deemed to be of poor quality, scores ranging from 4 to 6 were considered to be of moderate quality, and scores of 7 to 10 were considered to be of high quality [26].

The quality assessment was undertaken independently by two authors (M.P. and M.B.). The level of agreement was evaluated using kappa (κ) cut-off points recommended by Landis and Koch which divides into a poor agreement: < 0, slight agreement: 0–0.2, fair agreement: 0.2–0.4, moderate agreement: 0.4–0.6, substantial agreement: 0.6–0.8, and almost perfect agreement: 0.8–1.0 [27]. Any disagreements were discussed to reach a consensus. If no consensus was reached, a third researcher (H.N.) made the final decision.

### Data analysis

A random effects meta-analysis based on the DerSimonian and Laird model was performed for the primary outcomes due to expected methodological heterogeneity. The standardized mean difference (SMD) and 95% confidence intervals (CIs) were used as the effect size measure. The SMD was calculated using the mean and standard deviation (*i.e.,* Cohen’s *d* method) and was also interpreted according to Cohen’s rule of thumb: < 0.2: no/trivial effect; 0.2 to 0.5: small effect; > 0.5 to 0.8: medium effect; > 0.8: large effect [28]. Forest plots were used to display results from individual studies and pooled analyses. A negative effect size indicates that the differences in postural control parameters were in favor of asymptomatic participants. The secondary outcome is summarized narratively because of scarce data for pooling. It is worth noting that if a study included two groups of participants with LBP, we extracted data from the group with more severe symptoms to avoid duplicating comparisons. For example, in the study by Shanbehzadeh et al. [29] we specifically considered participants with chronic LBP and high pain-related anxiety for data extraction.

Statistical heterogeneity between the selected studies was assessed using the *I*^*2*^ statistic and Cochrane Q test with a significance level at *p* ≤ 0.05 [30]. The interpretation of *I*^*2*^ values was as follows: 0–40%: heterogeneity may not be important; 30%–60%: may represent moderate heterogeneity; 50–90%: may represent substantial heterogeneity and 75–100%: considerable heterogeneity [18].

Meta-analyses were conducted using the ‘metan’ package in Stata MP V.17.0 (StataCorp, College Station, Texas, USA). If data were not available in numerical format, we extracted it from figures using WebPlotDigitizer V.4.2 (https://automeris.io/WebPlotDigitizer/index.html).

### Subgroup analysis

Following a consensus-building meeting, the authors decided to stratify the analyses according to two levels of postural difficulty: easy (*i.e.*, rigid surface) and difficult (*i.e.*, foam surface or single leg stance). When at least two studies were available, we performed subgroup analyses based on different categories cognitive tasks. Bayot et al. proposed a classification of cognitive tasks including reaction time, discrimination and decision-making, mental tracking/working memory, and verbal fluency tasks [31]. Table 1 presents the definition of each category.

**Table 1:** A classification of cognitive tasks

Category	Definition
Reaction time	A type of task that measures the time between a sensory stimulus and a behavioral response
Discrimination and decision-making task	A type of that necessitates focused attention on a particular stimulus or feature and generating a specific response
Mental tracking/ working memory task	A type of task that demands the retention of information in the mind while potentially manipulating it through mental processes
Verbal fluency task	A type of task that involves generating words spontaneously or under predetermined search conditions. This task is commonly utilized to investigate executive function

### Assessment of publication bias

Given the limited number of studies included in the analysis, the Egger’s linear regression test [32] was employed for exploring publication bias. A significant level of 0.10 was adopted, since the number of studies was fewer than ten for each comparison.

## Results

### Study characteristics

**Fig. 1:**
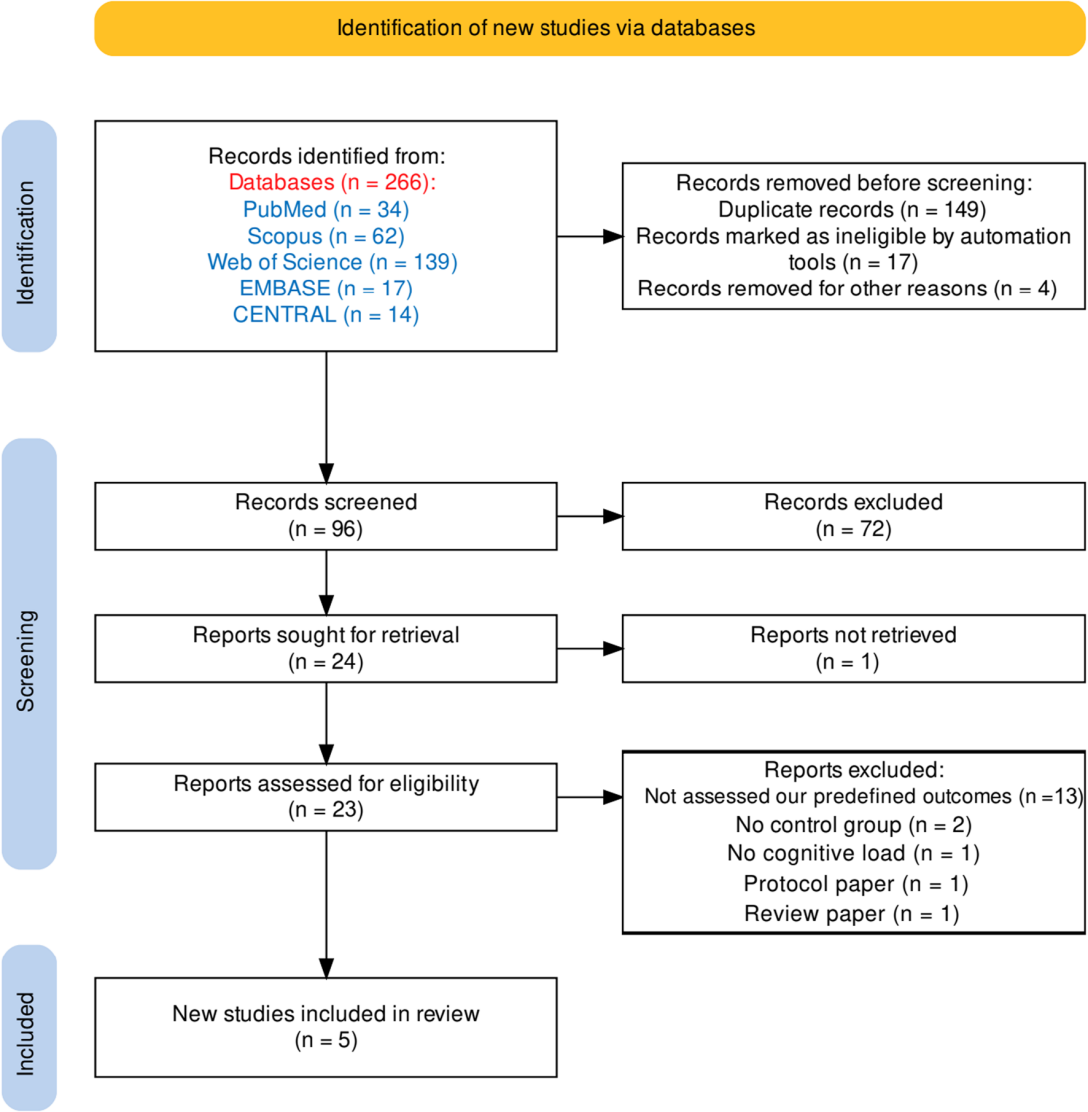
Flowchart of study selection. Flowchart adapted from the PRISMA 2020 statement

As of January 1, 2023, out of 266 initial studies, 23 were selected for full-text evaluation. However, 18 studies were excluded due to not meeting the predefined eligibility criteria [5, 33–49]. Specifically, 13 studies lacked at least one of the outcomes specified in our review [5, 33–44], two studies did not incorporate an asymptomatic control group [45, 46], one study did not incorporate a cognitive load [47], one study was identified as a protocol paper [48], and one study was identified as a review paper [49]. Furthermore, it should be noted that despite our efforts, we were unable to retrieve a study published in 1973 [50]. However, it is worth mentioning that the study was subsequently republished in 1991 [38]. The PRISMA flowchart of study selection is shown in Fig. 1. Four out of five studies included in this systematic review were conducted in the Iran [11, 14, 15, 29] and one study was performed in China [51]. All included studies were published in English and published between 2009 and 2021. Study characteristics are summarized in Table 2.

**Table 2:** Characteristics of the included studies in chronological order

Author	Journal	Country	Participants	Instrument	Experimental design	Category of cognitive task	Outcome
Ge et al. [49]	BMC Geriatrics	China	20 female patients with non-specific LBP for ≥ 3 months in the previous year, age ≥ 60 years (mea*n = *64.90 ± 3.33 y) and 20 healthy participants, age ≥ 60 years (mea*n = *63.20 ± 2.33 y)	TecnoBody system (PK254P; TecnoBody, Italy). All the center of pressure signals were sampled at a rate of 100 Hz and filtered at 8 Hz (by a 30th order low-pass FIR filter with zero-phase) and down-sampled at 20 Hz	Three postural tasks: (1) rigid-surface with eyes open, (2) rigid-surface with eyes closed, and (3) single leg stance *Data from tasks (1) and (3) were used for meta-analysis* Cognitive tasks: (1) no cognitive task, (2) auditory arithmetic task, and (3) serial-7s arithmetic task *Data from ‘serial-7s arithmetic’ task were used for meta-analysis*	Mental tracking/working memory tasks	Center of pressure parameters including sway area, sway length, AP velocity, and MD velocity
Shanbezadeh et al. [29]	Experimental Brain Research	Iran	38 patients with non-specific chronic LBP (19 with low and 19 with high pain-related anxiety levels) (mean age = 28.61 ± 1.33 y) and 20 asymptomatic participants (mean age = 28.32 ± 6.11 y)	Force platform (Bertec Corp, Columbus, OH, USA). Data were sampled at 500 Hz, and low-pass filtered with a cut-off frequency of 10 Hz	Four postural tasks: Eyes open with and without ankle vibration, eyes closed with and without ankle vibrations *Data from ‘eyes open without ankle vibration’ and ‘eyes closed with ankle vibrations’ tasks were used for meta-analysis* Cognitive tasks: (1) no auditory Stroop task, and (2) auditory Stroop task *Data from ‘auditory Stroop task’ task were used for meta-analysis*	Discrimination and decision-making tasks	Center of pressure parameters including area, velocity, AP range, and MD range; and reaction time, and error ratio of auditory Stroop test
Etemadi et al. [15]	Gait and Posture	Iran	20 patients with recurrent non-specific LBP (10 male, 10 female; mean age = 31.68 ± 8.63 y) and 20 asymptomatic participants (11 male, 9 female; mean age = 30.95 ± 8.08 y)	Force platform (Equitest, Neurocom Int. Inc., Clackamas, Oregon, USA). Data were recorded with a sampling rate of 500 Hz	Four postural tasks: (1) forward medium perturbation while standing, (2) forward large perturbation while standing, (3) backward medium perturbation while standing, and (4) backward large perturbation while standing *Data from ‘forward medium perturbation while standing’ and ‘backward large perturbation while standing’ tasks were used for meta-analysis* Cognitive tasks: (1) no auditory Stroop task, and (2) auditory Stroop task *Data from ‘auditory Stroop task’ task were used for meta-analysis*	Discrimination and decision-making tasks	Center of pressure parameters including amplitude, velocity, reaction time, latency, and reaction time for cognitive performance
Mazaheri et al. [11]	Spine	Iran	20 patients with current non-specific LBP (12 male, 8 female; mean age = 33.5 ± 9.2 y), 20 patients with recent non-specific LBP (12 male, 8 female; mean age = 35.3 ± 10.2 y), and 20 asymptomatic participants (12 male, 8 female; mean age = 34.3 ± 7.6 y)	A portable Kistler force platform (500 × 600 mm, type 9260 AA; Kistler Instruments, Winterthur, Switzerland). Data were sampled at a rate of 100 samples/second producing a total of 6000 COP data points for a recording of 60 s	Two postural tasks: (1) wide base of support, and (2) narrow base of support Cognitive tasks: (1) no color-number classification task, and (2) color-number classification task *Data from ‘color-number classification’ task were used for meta-analysis*	Discrimination and decision-making tasks	Center of pressure parameters including sway amplitude, path length, mean power frequency, and sample entropy
Salavati et al. [14]	Spine	Iran	22 patients with non-specific LBP (13 male, 9 female; mean age = 26.1 ± 6.2 y), and 22 asymptomatic participants (13 male, 9 female; mean age = 25 ± 5.5 y)	A strain gauge Bertec 4060–10 force platform and Bertec AM-6701 amplifier (Bertec Corp., Columbus, OH). Data were sampled at 200 Hz	Three postural tasks: (1) rigid-surface with eyes open, (2) rigid-surface with eyes closed, and (3) foam-surface with eyes closed *Data from ‘rigid-surface with eyes open’ and ‘foam-surface with eyes closed’ tasks were used for meta-analysis* Cognitive tasks: (1) easy task, and (2) difficult digits backward task *Data from ‘difficult digits backward’ task were used for meta-analysis*	Mental tracking/working memory tasks	Center of pressure parameters including velocity, phase plane portrait, and standard deviation of velocity

AP: anteroposterior; LBP: low back pain; MD: mediolateral

### Study population

The selected studies involved a total of 242 participants, consisting of 140 individuals with LBP and 102 without symptoms. The mean ages and standard deviations for both the patient and asymptomatic groups were 36.68 (14.21) and 36.35 (15.39) years, respectively. Of the included studies, three included both male and female participants [11, 14, 15], one study exclusively included female participants [51], and another study did not provide information on the gender of the study population [29]. All studies recruited participants with non-specific LBP (Table 2).

### Quality assessment in included studies

Table 3 presents a summary of the quality assessment of the reviewed articles. Of the five studies included in the analysis, three were classified as high-quality papers [11, 14, 51] while the remaining two [15, 29] were deemed to be of moderate quality. The two reviewers showed perfect agreement, with a weighted κ coefficient of 1. Four out of five studies did not provide a rationale for their sample size calculation [11, 14, 29, 51]. Furthermore, one study [11] appropriately matched the patient group and control group based on their level of physical activity.

**Table 3:** Quality assessment for each included study

Study	Selection				Comparability		Outcome measurement		Statistical analysis		Total score
	Patient definition^a^	Representativeness of patients^b^	Selection of controls^c^	Definition of controls^d^	Age and gender^e^	Physical activity^f^	Reliability of outcome measure^g^	Validity of outcome measure^h^	Sample size^i^	Statistical method^j^	
Salavati et al. [14]	**1**	**1**	**1**	**1**	**1**	*0*	**1**	**1**	*0*	**1**	8*
Mazaheri et al. [11]	**1**	**1**	**1**	**1**	**1**	**1**	**1**	**1**	*0*	**1**	9*
Etemadi et al. [15]	**1**	*0*	*0*	*0*	**1**	*0*	**1**	**1**	**1**	**1**	6
Shanbehzadeh et al. [29]	**1**	*0*	*0*	*0*	**1**	*0*	**1**	**1**	*0*	**1**	5
Ge et al. [49]	**1**	**1**	**1**	**1**	**1**	*0*	**1**	**1**	*0*	**1**	8*
Percentage of articles meeting each NOS item	100%	60%	60%	60%	100%	20%	100%	100%	20%	100%	

^*^Indicates high-quality studies

^a^The inclusion/exclusion criteria are clearly defined

^b^How were cases selected? (*e.g.*, random sample)

^c^Controls were derived from the same community as patients

^d^Controls defined as individuals with no history of low back pain

^e^The patient group and control group were adequately matched for age and gender

^f^The patient group and control group were adequately matched for physical activity

^g^The measure of postural control has documented reliability

^h^The measure of postural control has documented validity

^i^The sample size was justified

^j^The statistical analysis was clearly presented and was appropriate

## Main analyses

### Velocity *(n = 4)*

#### Easy postural control task and cognitive load

**Fig. 2:**
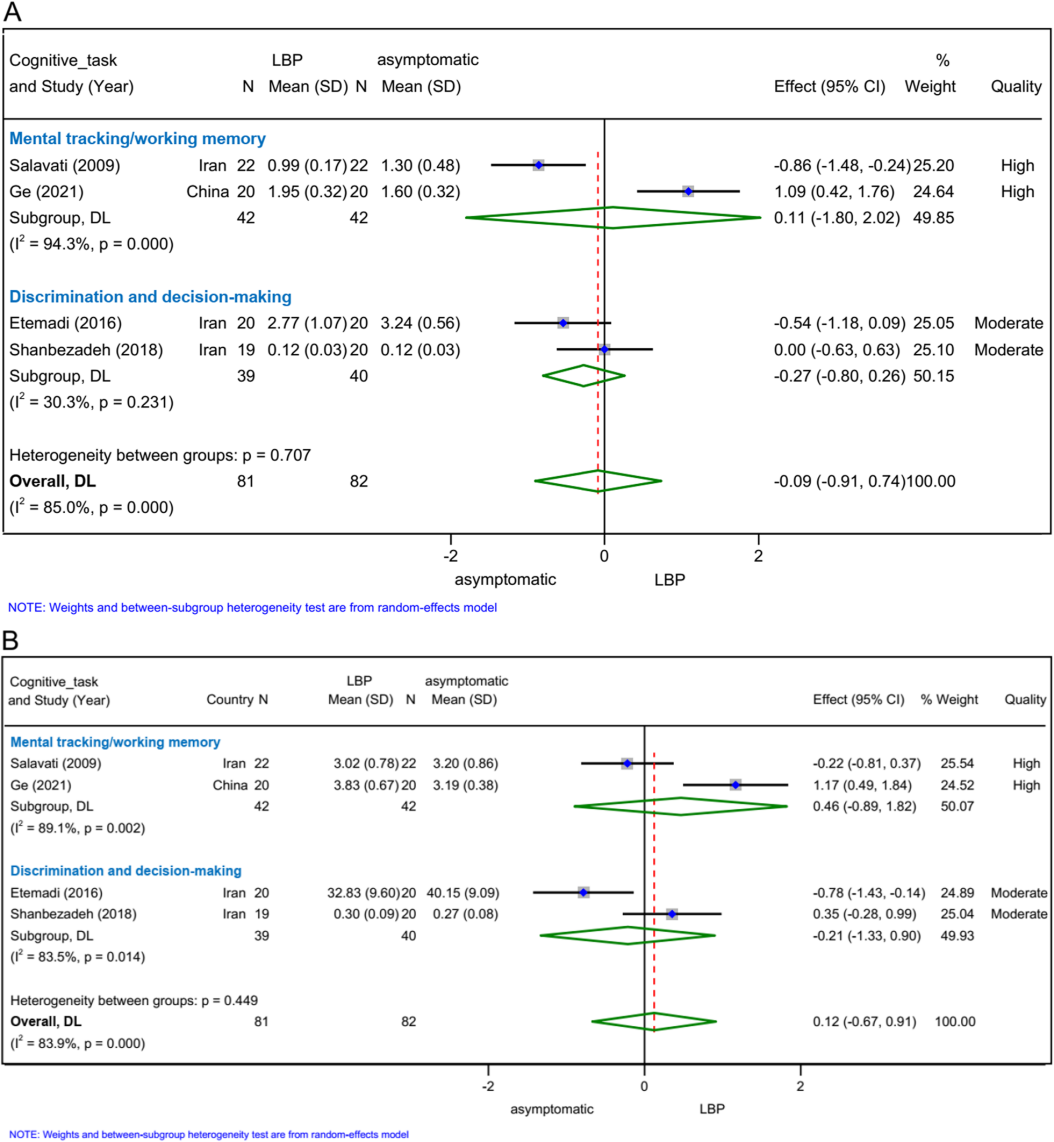
Forest plots for meta-analysis of velocity. **A** Easy postural control task and cognitive load, **B** difficult postural control task and cognitive load

Two high-quality studies [14, 51] and two moderate-quality studies [15, 29] assessed mean velocity during an easy postural control task and cognitive load. The meta-analysis suggested that there were no statistically significant differences between patients with and without LBP (SMD − 0.09, 95% CI − 0.91 to 0.74, *n = *163; Fig. 2A). In subgroup analysis conducted according to cognitive task, no difference between patients LBP and asymptomatic participants (mental tracking/working memory: SMD 0.11, 95% CI − 1.80 to 2.02, *n = *84; discrimination and decision-making: SMD − 0.27, 95% CI − 0.80 to 0.26, *n = *79; Fig. 2A) was either detected.

#### Difficult postural control task and cognitive load

The analysis of two high-quality studies [14, 51] and two moderate-quality studies [15, 29] showed no statistically significant difference in mean velocity between patients with LBP and asymptomatic participants during a difficult postural control task and cognitive load (SMD 0.12, 95% CI − 0.67 to 0.91, *n = *163; Fig. 2B). The results were not statistically or clinically changed in subgroup analysis stratified by cognitive task (mental tracking/working memory: SMD 0.46, 95% CI − 0.89 to 1.82, *n = *84; discrimination and decision-making: SMD − 0.21, 95% CI − 1.33 to 0.90, *n = *79; Fig. 2B).

### Area *(n = 4)*

**Fig. 3:**
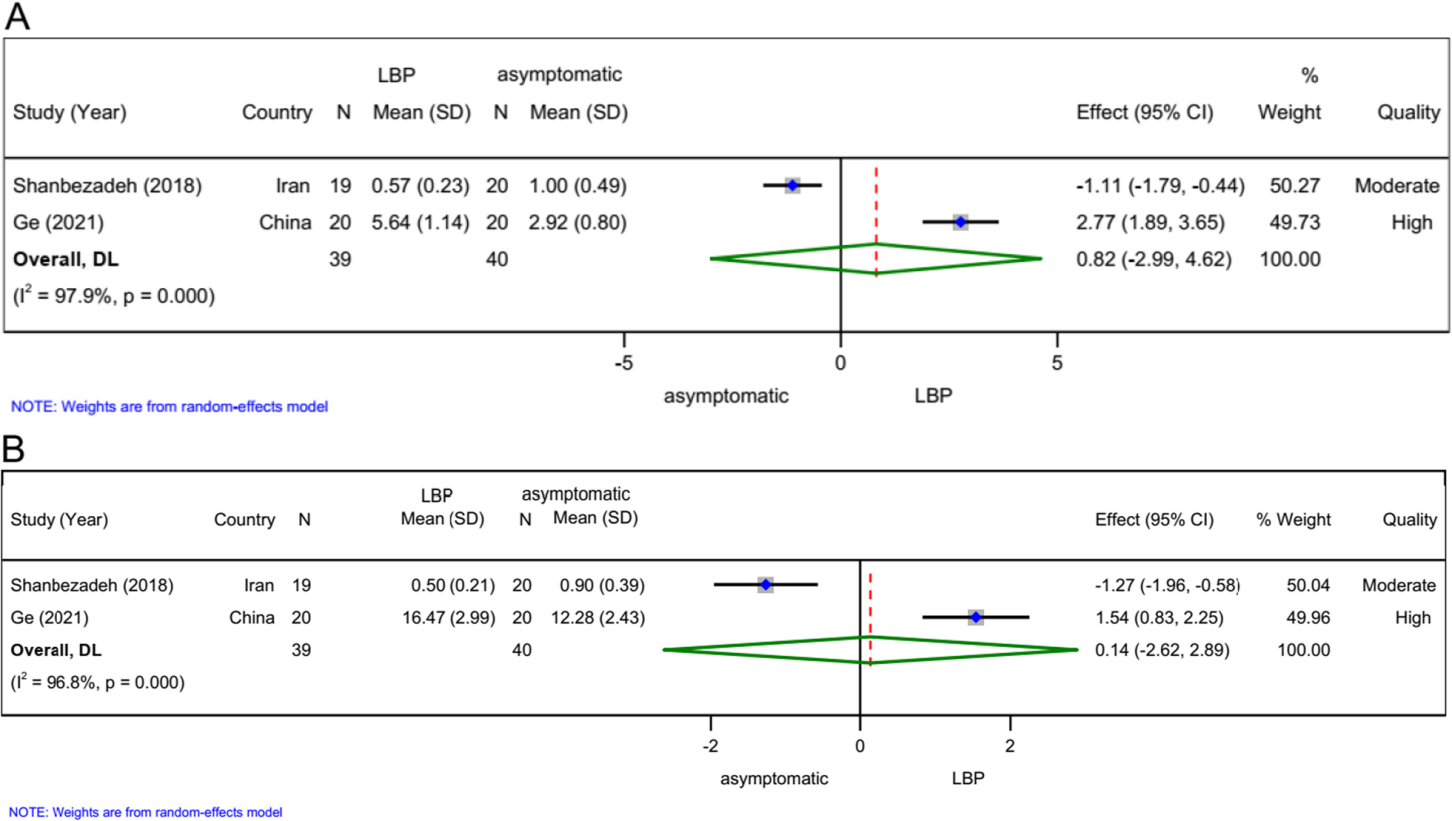
Forest plots for meta-analysis of area. **A** Easy postural control task and cognitive load, **B** difficult postural control task and cognitive load

The meta-analysis of a high-quality study [49] and a moderate-quality study [29] revealed no statistically between-groups differences after easy (SMD 0.82, 95% CI − 2.99 to 4.62, *n = *79; Fig. 3A) and difficult (SMD 0.14, 95% CI − 2.62 to 2.89, *n = *79; Fig. 3B) postural tasks and cognitive load.

### Phase plane portrait *(n = 1)*

In one high-quality study [14], no meaningful difference between patients with LBP and the asymptomatic population after either easy (SMD − 0.59, 95% CI − 1.19 to 0.02, *n = *44) or difficult (SMD − 0.18, 95% CI − 0.77 to 0.42, *n = *44) postural control task and cognitive load was observed.

### Path/sway length *(n = 2)*

**Fig. 4:**
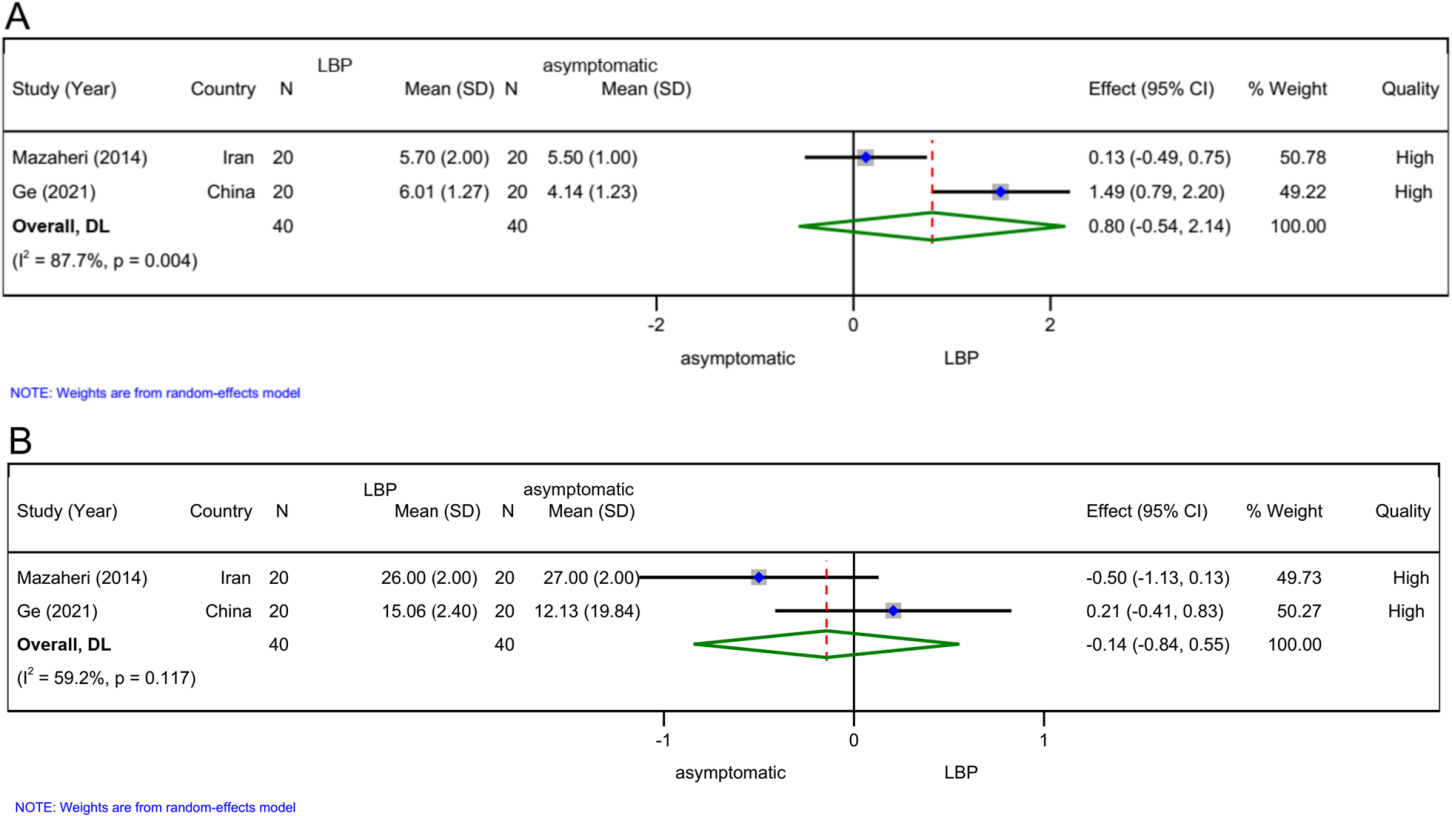
Forest plots for meta-analysis of path/sway length. **A** Easy postural control task and cognitive load, **B** difficult postural control task and cognitive load

In two high-quality studies [11, 51], the between-groups comparison revealed no significant difference after easy (SMD − 0.18, 95% CI − 0.77 to 0.42, *n = *80; Fig. 4A) or difficult (SMD − 0.14, 95% CI − 0.84 to 0.55, *n = *80; Fig. 4B) postural control task and cognitive load.

### Amplitude (*n = 2*)

**Fig. 5:**
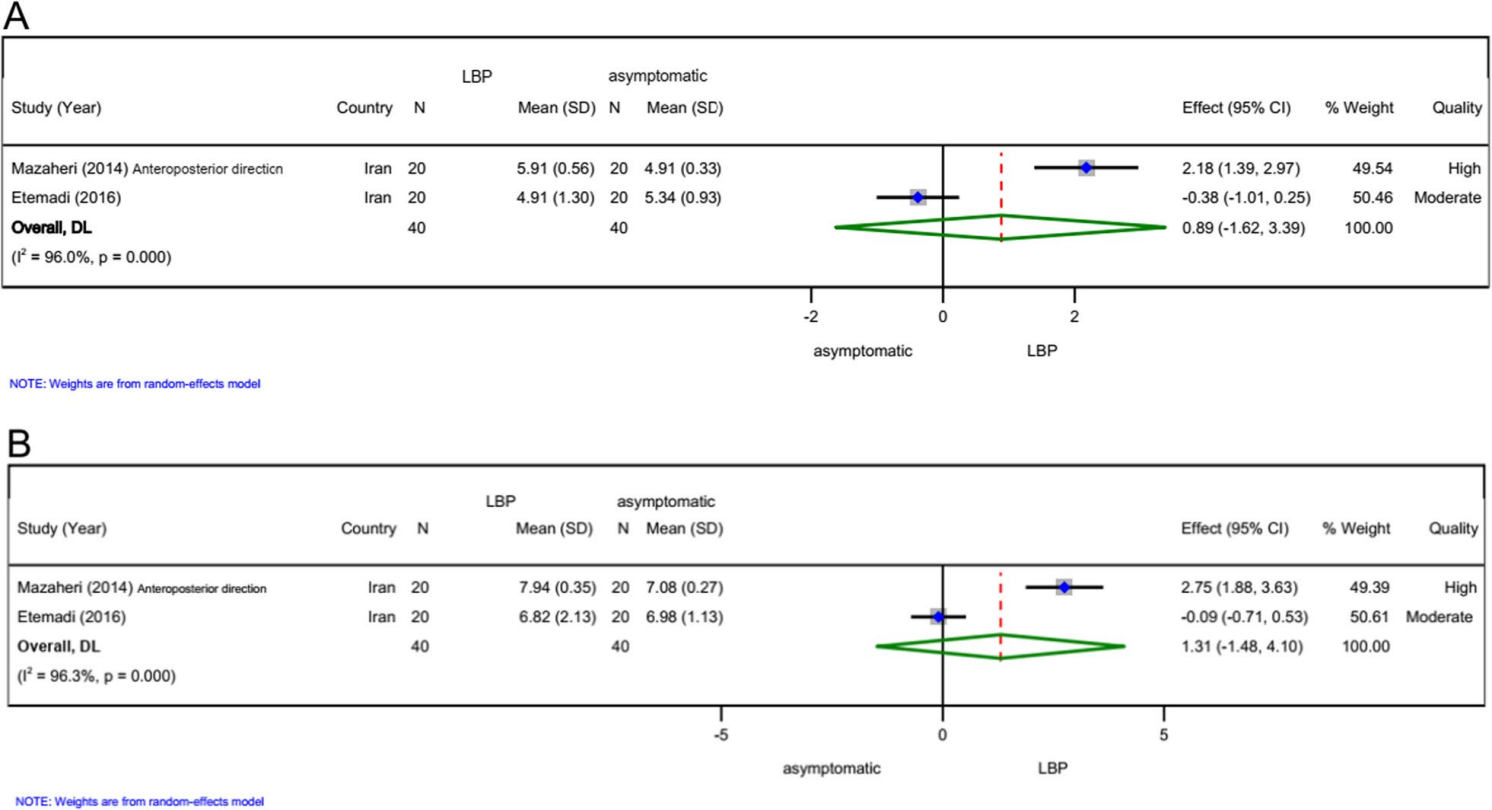
Forest plots for meta-analysis of amplitude. **A** Easy postural control task and cognitive load, **B** difficult postural control task and cognitive load

Two high-quality studies [11, 15] demonstrated that the amplitude of CoP did not statistically significant differ between patients with LBP and asymptomatic subjects after easy (SMD 0.89, 95% CI − 1.62 to 3.39, *n = *80; Fig. 5A) or difficult (SMD 1.31, 95% CI − 1.48 to 4.10, *n = *80; Fig. 5B) postural control task and cognitive load.

### Reaction time (*n = 2)*

A single study of moderate-quality [15] revealed that when performing an easy postural task with a cognitive load, individuals with non-specific LBP had slower reaction times compared to asymptomatic individuals (SMD 0.91, 95% CI 0.26 to 1.56, *n = *40). However, between-groups differences were not statistically significant when the postural task difficulty level was increased (SMD 0.53, 95% CI − 0.71 to 0.53, *n = *40). Furthermore, a moderate-quality study [29] revealed that individuals experiencing chronic LBP pain and heightened levels of pain-related anxiety displayed prolonged reaction times during both easy (SMD 2.00, 95% CI 1.22 to 2.78, *n = *39) and difficult (SMD 2.25, 95% CI 1.44 to 3.06, *n = *39) standing postural tasks, when compared to individuals without symptoms.

### Publication bias

The results of the Egger’s linear regression test revealed significant publication bias in the parameter velocity (bias 75.31, SE 20.56, 95% CI − 13.16 to 163.77; slope − 24.53, SE 6.67, 95% CI − 53.22 to 14.16, *P* = 0.07). Nevertheless, to arrive at a more comprehensive assessment of publication bias, further studies are necessary. Due to the limited data available, the trim-and-fill analysis could not be performed.

## Discussion

The objective of this systematic review and meta-analysis was to assess the impact of dual-task conditions on postural control among subjects with LBP compared to asymptomatic individuals. Based on the current literature, considering the methodological pitfalls identified, it seems that there is no significant difference in most postural control parameters between individuals with non-specific LBP and those without symptoms during dual-task activities. This finding challenges prior beliefs and underscores the significance of addressing methodological issues in future research to gain a more comprehensive understanding of this topic.

### Findings

Previous studies using a dual-task interference paradigm to investigate postural control in individuals with LBP have reported inconclusive results. Xiao et al. [17] proposed multiple factors that could affect postural control among patients with LBP when performing dual-task activities. However, there are two crucial factors that need to be considered in maintaining postural stability. The first factor is sensory strategies which have a significant role in maintaining postural stability [17]. When proprioceptive information from the ankle is reliable, individuals tend to rely on ankle strategy for postural control. This strategy entails a sequential activation pattern, starting with a delayed activation of ankle muscles, followed by the activation of thigh and trunk muscles in a distal-to-proximal manner [52]. In the presence of “small” challenges to postural stability, the ankle strategy acts as the body’s initial defense mechanism to counteract deviations in the CoP and prevent falls [52]. This could be one of the reasons why no significant difference in postural control during dual-task conditions was observed between individuals with and without LBP. In most of the included studies, the participants did not face significant challenges to postural stability, and therefore the CoP parameters did not need to endure extreme demands.

On the other hand, when the proprioceptive information from the ankle is unreliable, individuals rely more on back muscle information strategies to maintain posture [17]. Patients with LBP may experience a reduction or loss of back proprioceptive sensation, leading to decreased reliability of sensory information, which ultimately leads to poor performance in postural control [17]. In addition to proprioception, visual, vestibular, and auditory input also contribute significantly to sensory information processing. Inadequate sensory input may lead to adverse effects on postural control [17].

Another factor that influences postural control is the type and difficulty/complexity of cognitive tasks [51]. High cognitive demands can adversely affect postural control by competing with attentional resources that are needed for sensorimotor processing [53, 54]. Postural control is thought to depend on the difficulty of cognitive and motor tasks, and cognitive load has a bidirectional relationship with postural control [54]. Low cognitive load may promote postural control, but as cognitive load increases and resource competition becomes more intense, postural control can deteriorate [54]. Nevertheless, a recently published systematic review indicates that the use of cognitive tasks with varying complexity may not *fully* account for the varied effects observed in individual dual-task studies on postural stability in *healthy* populations, excluding other pathological conditions [55]. The review highlights that age and the level of difficulty in the postural task are critical factors contributing to this variability [55].

In addition to age, nature, and the complexity of postural tasks, other factors contribute to abnormal postural control, including cognitive resource capacity, attentional demands, complex sensorimotor integration, and the external environment [17]. The role of cognitive resources and higher cortical functions in maintaining postural stability is significantly impacted by participant age and the complexity of the postural task [55]. As attention resources, environmental conditions, and age-related cognitive functioning decline, the ability to maintain postural control generally decreases [17]. However, the results of our meta-analysis indicated that there were no significant differences observed in the postural control parameters measured between individuals with LBP and asymptomatic participants during dual-task conditions. The lack of difference could be attributed to several factors, such as the sample size being insufficient to detect significant differences between the groups. All studies included in our analysis, except for one [15], failed to calculate the sample size a priori. This may have led to a negative impact on the power of these studies. Additionally, the limited number of studies included in our analysis led to inconclusive results. Therefore, the findings of our meta-analysis should be interpreted with caution, and future studies may alter the conclusions derived from our analysis. It is also important to note that the findings of our study may not generalized to all individuals with LBP and may vary depending on the type, severity, and duration of the condition.

### Explanation of statistical heterogeneity

Our investigation revealed considerable heterogeneity among the studies reviewed. However, due to limited data availability, we refrained from conducting a meta-regression and a comprehensive sensitivity analysis. Despite this, the variability in the study settings and the methods used to evaluate the outcome of interest could offer plausible explanations for the observed heterogeneity in the results. Additionally, differences in study quality also appear to be a likely factor contributing to the considerable heterogeneity found in studies assessing the effect of dual-task conditions on postural control in patients with non-specific LBP. Only one study [11] out of the included studies matched the level of physical activity among the participants. The influence of physical activity on postural balance, as highlighted by Onofrei and Amaricai [56], may also be another source of the statistical heterogeneity. The population of participants with non-specific LBP included in these studies was not homogeneous, which may have further contributed to the observed variability in the results. Lastly, the differences in participants' age, gender, pain severity, and duration of LBP could introduce additional considerable heterogeneity.

### Comparison with previous systematic reviews

Although a direct comparison with a relevant systematic review is not available, recent evidence from a systematic review and meta-analysis has concluded that "cognitive task complexity may not determine whether postural stability increases or decreases during dual tasking […]" [55]. Moreover, our study did not find a statistically significant difference in postural control between individuals with non-specific LBP and asymptomatic participants. However, it is important to note that we cannot definitively conclude that cognitive task complexity has no impact on postural control in individuals with LBP, as the existing literature suggests a direct association between chronic LBP and cognitive dysfunction [57]. Therefore, considering the interactions between cognitive function and pain [58], the effects of cognitive task complexity on postural stability may vary for individuals with chronic LBP compared to the healthy population. Further studies are needed to obtain a comprehensive understanding of the effect of cognitive task complexity on postural control in patients with LBP.

## Limitations

The authors of this study have identified several limitations that need to be considered. Among these, the most notable are the limited number of high-quality studies and the ambiguity surrounding the influence of publication reporting bias. In our study, we utilized a selection criterion whereby only studies that reported at least one primary outcome that was pre-specified in our review were included. However, this criterion did not affect the number of studies included in our review, as none of the excluded studies measured the primary or secondary outcomes identified in our review. In addition, due to the small number of studies available on the topic, it was not feasible to conduct an extensive sensitivity analysis or meta-regression to identify potential reasons for the statistical heterogeneity observed in the existing literature. Consequently, the results of this systematic review lack conclusive evidence and should be approached with caution.

Our study did not find any significant differences between patients with LBP and asymptomatic participants regarding the impact of a cognitive task on postural control parameters during dual-task conditions. However, it is important to note that we did not specifically investigate how postural control demands affect cognitive functions in patients with LBP compared to asymptomatic participants. Stephan et al. [59] indicated that when individuals are required to simultaneously perform two tasks, the demands of maintaining postural control can influence how cognitive control is deployed. Specifically, it appears that in situations where two tasks need to be actively maintained in parallel, the level to which cognitive control enforces a more serial (shielded) mode versus a less selective attention mode that allows for more parallel processing of concurrently held active task rules can be affected by postural control demands. It is also noteworthy that the patients with non-specific LBP included in the studies were not homogeneous, as both chronic non-specific LBP and recurrent non-specific LBP were included. This heterogeneity may have impacted the results and the ability to draw definitive conclusions from the studies. Moreover, the cognitive tasks varied across the included studies, and no consistent use of a single cognitive task was present, which may have had differing effects on postural control. It has been demonstrated that cognitive tasks requiring more attentional resources, such as tasks with greater cognitive load, resulted in poorer postural control compared to tasks that required less attentional resources [60]. Hence, healthcare providers should take into account the type of cognitive task when designing rehabilitation programs for patients with non-specific LBP.

Moreover, it is necessary to acknowledge that three included studies had overlapping authorship [14, 15, 29], which introduces the potential for author bias. However, we conducted a rigorous quality assessment for each study to highlight the reliability and validity of the research.

It is worth noting that in this review, we did not include a comparison between single-task and dual-task performance in patients with non-specific LBP. Future systematic reviews could focus on comparing single-task and dual-task performance in individuals with LBP. Lastly, it is important to emphasize that our meta-analysis was based on aggregated study-level data rather than individual participant data.

## Recommendations for future research

The current body of literature on the impact of dual-task conditions on postural control in patients with LBP exhibits a noticeable gap. To fill this void, there is a need for high-quality research that employs transparent methodology to enhance the degree of certainty regarding the aforementioned impact. It is crucial for forthcoming investigations to perform a priori sample size estimation to ensure the statistical power of their findings. Additionally, clinical trials can evaluate the long-term effectiveness of dual-task conditions on postural control parameters among patients with LBP.

## Conclusion

The results observed in this review suggest that there is no significant difference in postural control during dual-task conditions between individuals with non-specific LBP and those without pain. The analysis of multiple outcomes such as velocity, area, phase plane, path/sway length, and amplitude did not reveal any significant differences between groups. Although one moderate-quality study showed that individuals with non-specific LBP had slower reaction times during an easy postural task with a cognitive load, the difference was not statistically significant when the postural task difficulty level was increased. Another moderate-quality study revealed that individuals experiencing chronic non-specific LBP and heightened levels of pain-related anxiety displayed prolonged reaction times during both easy and difficult standing postural tasks, when compared to those without symptoms. However, it is important to acknowledge that the meta-analysis was conducted based on a restricted pool of studies featuring small sample sizes. Additionally, the presence of publication reporting bias and considerable statistical heterogeneity further adds complexity to the interpretation of the results. Therefore, it is of utmost importance to exercise extreme caution when drawing conclusions from these findings. Consequently, to establish definitive conclusions, it is imperative to conduct further high-quality studies with larger sample sizes that can yield more statistically robust findings.

## Supplementary Information

**Additional file 1. **Search Terms and Strategies.

## Data Availability

The data and materials used and/or analyzed during the current study are available from the corresponding author on reasonable request.

## Abbreviations

CI: Confidence interval
CoP: Center of pressure
LBP: Low back pain
MeSH: Medical subject headings
NOS: Newcastle–Ottawa Scale
PECO: Population, exposure, comparator, outcome
PRESS: Peer Review of Electronic Search Strategies
PRISMA: Preferred Reporting Items for Systematic Reviews and Meta-Analyses
SMD: Standardized mean difference
