# Supplementary material for: The effect of dual-task conditions on postural control in adults with low back pain: a systematic review and meta-analysis
Source: J Orthop Surg Res. 2023 Aug 1;18:555. doi: 10.1186/s13018-023-04035-6 (PMC10391969; doi:10.1186/s13018-023-04035-6)
Supplement: Supplementary file 1 — Additional file 1. Search Terms and Strategies. [file 13018_2023_4035_MOESM1_ESM.docx]

**Supplementary Information**

**SEARCH TERMS AND STRATEGIES**

**Population:**

**Search for PubMe****d**

**Exposure (E component):** ((‘dual task’[TIAB] OR ‘dual tasks’[TIAB] OR ‘double task’[TIAB] OR ‘double tasks’[TIAB] OR ‘multiple task’[TIAB] OR ‘multiple tasks’[TIAB] OR ‘secondary task’[TIAB] OR ‘secondary tasks’[TIAB] OR ‘second task’[TIAB] OR ‘second tasks’[TIAB] OR ‘two task’[TIAB] OR ‘two tasks’[TIAB] OR ‘cognitive task’[TIAB] OR ‘cognitive tasks’[TIAB] OR ‘cognitive load’[TIAB] OR ‘cognitive loads’[TIAB] OR ‘double assignment’[TIAB] OR ‘double assignments’[TIAB] OR ‘cognitive interference’[TIAB] OR ‘cognitive interferences’[TIAB] OR ‘cognitive performance’[TIAB] OR attention*[TIAB] OR ‘reaction time’[TIAB] OR ‘reaction times’[TIAB])

AND

**Outcome (O component):** (‘postural control’[TIAB] OR ‘postural controls’[TIAB] OR ‘postural stability’[TIAB] OR ‘postural stabilities’[TIAB] OR ‘postural balance’[TIAB] OR ‘postural balances’[TIAB] OR ‘postural equilibrium’[TIAB] OR ‘postural equilibriums’[TIAB] OR ‘postural sway’[TIAB] OR ‘postural sways’[TIAB] OR ‘postural performance’[TIAB] OR posturography[TIAB] OR posturographies[TIAB] OR stabilometry[TIAB] OR stabilometries[TIAB] OR baropodometry[TIAB] OR baropodometries[TIAB] OR ‘balance control’[TIAB] OR ‘balance controls’[TIAB] OR ‘center of pressure’[TIAB] OR center-of-pressure[TIAB] OR COP[TIAB] OR ‘centre of pressure’[TIAB] OR centre-of-pressure[TIAB] OR ‘balance biodex’[TIAB] OR ‘static balance’[TIAB] OR ‘dynamic balance’[TIAB] OR ‘postural interference’[TIAB] OR ‘postural interferences’[TIAB] OR ‘postural recovery’[TIAB])

AND

**Population (P component):** ((‘back pain’[TIAB] AND low*[ TIAB]) OR (‘back pains’[TIAB] AND low*[TIAB]) OR (‘low back’[TIAB] AND pain*[TIAB]) OR lumbago[TIAB] OR (‘lower back’[TIAB] AND pain*[TIAB]) OR (‘back ache’[TIAB] AND low*[TIAB]) OR (‘back aches’[TIAB] AND low*[TIAB]) OR (low[TIAB] AND backache*[TIAB]) OR (‘low back pain’[TIAB] AND ‘posterior compartment’[TIAB]) OR (‘low back pain’[TIAB] AND recurrent[TIAB]) OR (‘low back pain’[TIAB] AND mechnical[TIAB]) OR ‘lumbalgia’[TIAB] OR ‘back disorder’[TIAB] OR (low-back[TIAB] AND pain*[TIAB]))

**N = 34**

**Search Strategy for Scopus**

**Exposure (E component):** TITLE-ABS-KEY((“dual task” OR “dual tasks” OR “double task” OR “double tasks” OR “multiple task” OR “multiple tasks” OR “secondary task” OR “secondary tasks” OR “second task” OR “second tasks” OR “two task” OR “two tasks” OR “cognitive task” OR “cognitive tasks” OR “cognitive load” OR “cognitive loads” OR “double assignment” OR “double assignments” OR “cognitive interference” OR “cognitive interferences” OR “cognitive performance” OR attention* OR “reaction time” OR “reaction times”))

AND

**Outcome (O component):** TITLE-ABS-KEY(( “postural control” OR “postural controls” OR “postural stability” OR “postural stabilities” OR “postural balance” OR “postural balances” OR “postural equilibrium” OR “postural equilibriums” OR “postural sway” OR “postural sways” OR “postural performance” OR posturography OR posturographies OR stabilometry OR stabilometries OR baropodometry OR baropodometries OR “balance control” OR “balance controls” OR “center of pressure” OR center-of-pressure OR COP OR “centre of pressure” OR centre-of-pressure OR “balance biodex” OR “static balance” OR “dynamic balance” OR “postural interference” OR “postural interferences” OR “postural recovery”))

AND

**Population (P component):** TITLE-ABS-KEY(((“back pain” AND low*) OR (“back pains” AND low*) OR (“low back” AND pain*) OR lumbago OR (“lower back” AND pain*) OR (“back ache” AND low*) OR (“back aches” AND low*) OR (low AND backache*) OR (“low back pain” AND “posterior compartment”) OR (“low back pain” AND recurrent) OR (“low back pain” AND mechnical) OR “lumbalgia” OR “back disorder” OR (low-back AND pain*)))

Document Type: Article

Source Type: Journal

**N = 62**

**Search Strategy for WoS**

**Exposure (E component):** Ts=((“dual task” OR “dual tasks” OR “double task” OR “double tasks” OR “multiple task” OR “multiple tasks” OR “secondary task” OR “secondary tasks” OR “second task” OR “second tasks” OR “two task” OR “two tasks” OR “cognitive task” OR “cognitive tasks” OR “cognitive load” OR “cognitive loads” OR “double assignment” OR “double assignments” OR “cognitive interference” OR “cognitive interferences” OR “cognitive performance” OR attention* OR “reaction time” OR “reaction times”))

AND

**Outcome (O component):** Ts=(( “postural control” OR “postural controls” OR “postural stability” OR “postural stabilities” OR “postural balance” OR “postural balances” OR “postural equilibrium” OR “postural equilibriums” OR “postural sway” OR “postural sways” OR “postural performance” OR posturography OR posturographies OR stabilometry OR stabilometries OR baropodometry OR baropodometries OR “balance control” OR “balance controls” OR “center of pressure” OR center-of-pressure OR COP OR “centre of pressure” OR centre-of-pressure OR “balance biodex” OR “static balance” OR “dynamic balance” OR “postural interference” OR “postural interferences” OR “postural recovery”))

AND

**Population (P component):** Ts=(((“back pain” AND low*) OR (“back pains” AND low*) OR (“low back” AND pain*) OR lumbago OR (“lower back” AND pain*) OR (“back ache” AND low*) OR (“back aches” AND low*) OR (low AND backache*) OR (“low back pain” AND “posterior compartment”) OR (“low back pain” AND recurrent) OR (“low back pain” AND mechnical) OR “lumbalgia” OR “back disorder” OR (low-back AND pain*)))

Document Types: Article

**N = 139**

**Search for Embase**

**Exposure (E component):** (‘dual task’:ti,ab OR ‘dual tasks’:ti,ab OR ‘double task’:ti,ab OR ‘double tasks’:ti,ab OR ‘multiple task’:ti,ab OR ‘multiple tasks’:ti,ab OR ‘secondary task’:ti,ab OR ‘secondary tasks’:ti,ab OR ‘second task’:ti,ab OR ‘second tasks’:ti,ab OR ‘two task’:ti,ab OR ‘two tasks’:ti,ab OR ‘cognitive task’:ti,ab OR ‘cognitive tasks’:ti,ab OR ‘cognitive load’:ti,ab OR ‘cognitive loads’:ti,ab OR ‘double assignment’:ti,ab OR ‘double assignments’:ti,ab OR ‘cognitive interference’:ti,ab OR ‘cognitive interferences’:ti,ab OR ‘cognitive performance’:ti,ab OR attention*:ti,ab OR ‘reaction time’:ti,ab OR ‘reaction times’:ti,ab)

AND

**Outcome (O component):** ( ‘postural control’:ti,ab OR ‘postural controls’:ti,ab OR ‘postural stability’:ti,ab OR ‘postural stabilities’:ti,ab OR ‘postural balance’:ti,ab OR ‘postural balances’:ti,ab OR ‘postural equilibrium’:ti,ab OR ‘postural equilibriums’:ti,ab OR ‘postural sway’:ti,ab OR ‘postural sways’:ti,ab OR ‘postural performance’:ti,ab OR posturography:ti,ab OR posturographies:ti,ab OR stabilometry:ti,ab OR stabilometries:ti,ab OR baropodometry:ti,ab OR baropodometries:ti,ab OR ‘balance control’:ti,ab OR ‘balance controls’:ti,ab OR ‘center of pressure’:ti,ab OR center-of-pressure:ti,ab OR COP:ti,ab OR ‘centre of pressure’:ti,ab OR centre-of-pressure:ti,ab OR ‘balance biodex’:ti,ab OR ‘static balance’:ti,ab OR ‘dynamic balance’:ti,ab OR ‘postural interference’:ti,ab OR ‘postural interferences’:ti,ab OR ‘postural recovery’:ti,ab)

AND

**Population (P component):** ((‘back pain’:ti,ab AND low*:ti,ab) OR (‘back pains’:ti,ab AND low*:ti,ab) OR (‘low back’:ti,ab AND pain*:ti,ab) OR lumbago:ti,ab OR (‘lower back’:ti,ab AND pain*:ti,ab) OR (‘back ache’:ti,ab AND low*:ti,ab) OR (‘back aches’:ti,ab AND low*:ti,ab) OR (low:ti,ab AND backache*:ti,ab) OR (‘low back pain’:ti,ab AND ‘posterior compartment’:ti,ab) OR (‘low back pain’:ti,ab AND recurrent:ti,ab) OR (‘low back pain’:ti,ab AND mechnical:ti,ab) OR ‘lumbalgia’:ti,ab OR ‘back disorder’:ti,ab OR (low-back:ti,ab AND pain*:ti,ab))

**N = 17**

**Search for the Cochrane Central Register of Controlled Trials**

**Exposure (E component):** ((“dual task” OR “dual tasks” OR “double task” OR “double tasks” OR “multiple task” OR “multiple tasks” OR “secondary task” OR “secondary tasks” OR “second task” OR “second tasks” OR “two task” OR “two tasks” OR “cognitive task” OR “cognitive tasks” OR “cognitive load” OR “cognitive loads” OR “double assignment” OR “double assignments” OR “cognitive interference” OR “cognitive interferences” OR “cognitive performance” OR attention* OR “reaction time” OR “reaction times”)

AND

**Outcome (O component):** ( “postural control” OR “postural controls” OR “postural stability” OR “postural stabilities” OR “postural balance” OR “postural balances” OR “postural equilibrium” OR “postural equilibriums” OR “postural sway” OR “postural sways” OR “postural performance” OR posturography OR posturographies OR stabilometry OR stabilometries OR baropodometry OR baropodometries OR “balance control” OR “balance controls” OR “center of pressure” OR center-of-pressure OR COP OR “centre of pressure” OR centre-of-pressure OR “balance biodex” OR “static balance” OR “dynamic balance” OR “postural interference” OR “postural interferences” OR “postural recovery”)

AND

**Population (P component):** ((“back pain” AND low*) OR (“back pains” AND low*) OR (“low back” AND pain*) OR lumbago OR (“lower back” AND pain*) OR (“back ache” AND low*) OR (“back aches” AND low*) OR (low AND backache*) OR (“low back pain” AND “posterior compartment”) OR (“low back pain” AND recurrent) OR (“low back pain” AND mechnical) OR “lumbalgia” OR “back disorder” OR (low-back AND pain*)) in Title Abstract Keyword

**N = 14**
